# Atg5-Deficient Mice Infected with *Francisella tularensis* LVS Demonstrate Increased Survival and Less Severe Pathology in Internal Organs

**DOI:** 10.3390/microorganisms8101531

**Published:** 2020-10-06

**Authors:** Ina Kelava, Mirna Mihelčić, Mateja Ožanič, Valentina Marečić, Maša Knežević, Marija Ćurlin, Sanja Štifter, Anders Sjöstedt, Marina Šantić

**Affiliations:** 1 Department of Microbiology and Parasitology, University of Rijeka, Faculty of Medicine, 51000 Rijeka, Croatia; ina.kelava@medri.uniri.hr (I.K.); mirna.mihelcic@medri.uniri.hr (M.M.); mateja.ozanic@medri.uniri.hr (M.O.); valentina.marecic@medri.uniri.hr (V.M.); masa.knezevic@medri.uniri.hr (M.K.); 2 Department of Histology and Embryology, Faculty of Medicine, University of Zagreb, 10000 Zagreb, Croatia; marija.curlin@mef.hr; 3 Department of Pathology, Faculty of Medicine, University of Rijeka, 51000 Rijeka, Croatia; sanja.stifter@medri.uniri.hr; 4 Department of Clinical Microbiology, Umeå University, 901 85 Umeå, Sweden; anders.sjostedt@umu.se

**Keywords:** *Francisella*, Atg5, autophagy, mice

## Abstract

*Francisella tularensis* is a highly virulent intracellular pathogen that proliferates within various cell types and can infect a multitude of animal species. *Francisella* escapes the phagosome rapidly after infection and reaches the host cell cytosol where bacteria undergo extensive replication. Once cytosolic, *Francisella* becomes a target of an autophagy-mediated process. The mechanisms by which autophagy plays a role in replication of this cytosolic pathogen have not been fully elucidated. In vitro, *F. tularensis* avoids degradation via autophagy and the autophagy process provides nutrients that support its intracellular replication, but the role of autophagy in vivo is unknown. Here, we investigated the role of autophagy in the pathogenesis of tularemia by using transgenic mice deficient in Atg5 in the myeloid lineage. The infection of Atg5-deficient mice with *Francisella tularensis* subsp. *holarctica* live vaccine strain (LVS) resulted in increased survival, significantly reduced bacterial burden in the mouse organs, and less severe histopathological changes in the spleen, liver and lung tissues. The data highlight the contribution of Atg5 in the pathogenesis of tularemia in vivo.

## 1. Introduction

Intracellular bacterial pathogens use various strategies to survive and replicate within the host cells [1,2]. One of the strategies used is to escape from the degradative endocytic compartment into the host cytosol. When intracellular pathogens reach the cytosol, they become the targets of innate immune recognition via the autophagy pathway [3]. However, some intracellular pathogens, such as *Mycobacterium tuberculosis*, are eliminated efficiently via autophagy after the fusion of an autophagosome and a lysosome (autolysosome) [4]. In contrast, the cytosolic pathogens as *Shigella flexneri* and *Listeria monocytogenes* avoid autophagy and survive in the cell by secreting a specific protein on the bacterial surface that interferes with the autophagy cascade, or by masking themselves from autophagy recognition (recruitment of host proteins) [5,6,7]. The autophagy pathway removes the cytoplasmic material, such as damaged organelles and misfolded proteins and detects and eliminates invading cytosolic pathogens by producing autophagosomes/autolysosomes. Therefore, autophagy is essential for the homeostasis and the host’s ability to respond to the infection caused by an intracellular pathogen [8,9]. It is known that macroautophagy can occur through at least two different pathways. The canonical autophagy pathway is mainly induced by the inhibition of mammalian target of rapamycin (mTOR). The protein Atg5 is essential for the formation of a double-membrane vacuole, the autophagosome in the canonical autophagy pathway [10]. A lot of cytosolic pathogens are targets for canonical autophagy after binding to polyubiquitin and p62/SQSTM1 [6,11,12]. For pathogens such as *Streptococcus pyogens*, *Shigella flexneri*, *Salmonella*, and *Listeria*, the autophagy mechanism is dependent on Atg5 [7,13,14,15]. However, cells lacking essential genes such as Atg5 can still form autolysosomes by Atg5-independent autophagy. Unlike canonical macroautophagy, Atg5-independent autophagosomes are generated from the *trans*-Golgi and late endosomes [16]. For example, Atg5 is not required for autophagy in the lifecycles of *Mycobacterium marinum* and *Brucella abortus* [17,18].

*Francisella tularensis* is a highly virulent intracellular pathogen and is the causative agent of tularemia in humans and animals [19,20]. Three subspecies of *F. tularensis* subsp. *tularensis*, subsp. *holarctica*, and subsp. *mediasiatica* can cause tularemia in humans [21]. The *F. tularensis* live vaccine strain (LVS) has been derived from *F. tularensis* subsp. *holarctica*. The LVS is attenuated for humans, but highly virulent for mice. Accordingly, the LVS has been often used as a model organism to study the pathogenesis of tularemia and the virulence mechanisms of *F. tularensis* [22,23,24].

Essential to the development of tularemia, *Francisella* proliferates within various cell types including macrophages, hepatocytes, pneumocytes, and dendritic cells [25]. It is known that macrophages are a key niche for the survival and the replication of *F. tularensis*. Due to its importance in the intracellular trafficking, many studies have been focused on the lifecycle of *F. tularensis* within macrophages [26]. Previous studies have shown that after the phagocytic uptake, *Francisella* resides inside a phagosomal vacuole (FCP, *Francisella*-containing phagosome). Early after the infection (30–60 min), *Francisella* strains escape the phagosome into the macrophage cytosol where the bacteria undergo extensive replication. Due to its rapid phagosomal escape, *Francisella* interacts with the endocytic pathway early after the infection [26,27,28,29,30]. At the postreplication stage, bacteria re-enter the double-membrane vacuole via an autophagy-mediated process [31,32,33]. It has been shown that the formation of the *Francisella*-containing vacuole (FCV) is dependent on an autophagy process and it occurs between 20 and 30 h after the infection [31]. Bacterial degradation was not observed in most of the FCVs, indicating that *Francisella* can avoid a selective degradation of cytosolic pathogens via an autophagy pathway known as xenophagy [34]. Additionally, a previous study has shown that FCVs are not involved in the intracellular replication and killing, but they require autophagy for their formation and display autophagy features [31]. 

Previous in vitro studies showed that the treatment of *F. tularensis* with autophagy inhibitors, such as chloroquine or ammonium chloride, impairs its intracellular replication [35,36,37]. Furthermore, it was shown that within Atg5^−/−^ macrophages, *Francisella* replicates efficiently [11]. Additionally, a study on the J774A.1 macrophage-like cells, mouse embryonic fibroblasts (MEFs), and human monocyte-derived macrophages (hMDMs) showed that *Francisella* not only avoids degradation via autophagy, but also provide nutrients that support its intracellular replication via an Atg5-independent autophagy mechanism, which is not associated with polyubiquitin, p62/SQSTM1, or LC3B [38]. Other studies showed that the O-antigen on the bacterial surface has a protective role against autophagy. They showed that O-antigen mutants are killed via Atg5-dependent autophagy after ubiquitination, indicating that the bacteria without O-antigens would be rapidly targeted by an autophagy pathway in the cytosol [39].

Although the previous studies demonstrated an important role of autophagy for the exacerbating intracellular replication of *F. tularensis* in in vitro models, the role of autophagy in vivo is unknown. Herein, we demonstrate that mice deficient in the Atg5-myeloid lineage show increased survival and less severe organ pathology upon infection with *F. tularensis* LVS.

## 2. Materials and Methods

### 2.1. Bacterial Strain

The *F. tularensis* subsp. *holarctica* LVS (Live Vaccine Strain) was kindly obtained from prof. Anders Sjöstedt (Umeå University, Umeå, Sweden). The strain was grown on a gonococci agar base (BD 228950, Fischer Scientific, Pittsburgh, PA, USA) supplemented with IsoVitaleX (BD 211876, Fischer Scientific, Pittsburgh, PA, USA) at 37 °C with 5% CO_2_ for 48 h.

### 2.2. Mice

The C57BL/6 *Atg5^flox/flox^* mice (strain: B6.129S-Atg5<tm1Myok>) were purchased form RIKEN BioResource Research Center, Japan. Lys2Mcre mice (strain: B6.129P2-Lyz2^tm1(cre)/fo^/J) were purchased form the Jackson Laboratory, Bar Harbor, USA. All animals were bred and housed in the animal facility of the Faculty of Medicine, University of Rijeka according to the Institutional and National guidelines. The C57BL/6 *Atg5^flox/^*^flox^ mice were crossed with Lys2Mcre mice to obtain myeloid cell-specific Atg5-deficient C57BL/6 *Atg5^flox/flox^*-*Lyz-Cre* mice (designated Atg5-deficient mice). C57BL/6 *Atg5^flox/^*^flox^ mice were used as a control. Mice which were 8–9 weeks old were used in all of the experiments. All mouse handling and experimental methods were conducted under the recommendation of the 3R law regulation procedure of the Republic of Croatia. The Ethic Committee approval number is 003-08/17-01/06.

### 2.3. Genotyping

The C57BL/6 *Atg5^flox/flox^*-*Lyz-Cre* mice genotypes were determined via PCR analysis of DNA from tail biopsies to confirm that the Atg5 fragment was deleted, as previously described [40]. The DNA of the C57BL/6 *Atg5^flox/^*^flox^ mice and/or Lys2Mcre mice were used as positive controls in the PCR analysis. To detect wild-type Atg5 and Atg5^flox^ alleles, we used following PCR primers: ATG5 exon 3-1: 5’-GAATATGAAGGCACACCCCTGAAATG-3’; ATG5 check 2: 5’-CAACGTCGAGCACAGCTGCGCAAGG-3’; ATG5 short 2: 5’- GTACTGCATAATGGTTTAACTCTTGC-3’; oIMR3066: 5’-CCCAGAAATGCCAGATTACG-3’; oIMR3067: 5’-CTTGGGCTGCCAGAATTTCTC-3’; oIMR3068: 5’-TTACAGTCGGCCAGG CTG AC-3’.

### 2.4. Infection Procedure, Mortality and Bacterial Load in Organs

For the survival assay, 10 C57BL/6 *Atg5^flox/flox^*-*Lyz-Cre* mice per group and 10 control mice were infected intradermally with the LVS in a dose of 5 × 10^4^, 5 × 10^5^, 5 × 10^6^, 5 × 10^7^, or 5 × 10^8^ bacteria, respectively. Infected mice were monitored daily during fifteen days. The mean time to death (MTTD) of two groups of mice was calculated corresponding to a survival assay. Based on the survival assay results, in all subsequent experiments, the C57BL/6 *Atg5^flox/flox^*-*Lyz-Cre* mice and the control mice were infected intradermally with the LVS in a dose of 5 × 10^4^ bacteria per mouse. Three mice per group were used in the rest of the experiment. Actual concentrations of the inoculum were determined by plating 10-fold serial dilutions on the agar plate. At 6, 20, 48 and 72 h after infection, the mice were induced in deep anesthesia, perfused and euthanized. To determine the bacterial loads in the lung, liver, and spleen, the organs were harvested and homogenized in 5 mL of sterile saline. Following the homogenization, the cells were lysed in distilled water. The number of bacteria was determined by plating serial 10-fold dilutions on modified gonococci agar at 37 °C with 5% CO_2_ for 48 h. 

### 2.5. Histopathology

For histopathology, at 20, 48, and 72 h after infection, three mice per group were humanly euthanized. The pulmonary vasculature was perfused with 10 mL of saline via the right ventricle. Following perfusion, the liver, lung, and spleen of each group of the infected mice were harvested aseptically, formalin-fixed and/or frozen in liquid nitrogen using Tissue Tek (Sakura Finetek, Torrance, CA, USA). Serial sections (5 µm) were stained with hematoxylin and eosin (H&E), covered with coverslips and prepared for the light microscopy analyses. The slides were scanned with the Hamamatsu slide scanner at ×20 HPF (Hamamatsu S60, Herrsching am Ammersee, Germany). The semi-quantitative analysis was performed on the digitalized slides. The observed morphological changes were assessed and the histopathology scoring was performed at twenty random high-power fields (HPFs) to grade the severity of the inflammation as well as the overall pathological characteristics of the spleen, liver and lung parenchyma. The liver tissue was scored for a hepatocyte vacuolation and inflammatory infiltrates (0—absent, 1—slight, 2—moderate, 3—severe). The spleen tissue was scored for the destruction of the architecture of red pulp, white pulp and capsule, and for the destruction of the spleen tissue (0—absent, 1—slight, 2—moderate, 3—severe). The lung tissue was scored for the alveolar and bronchial destruction, and infiltration of mononuclear cells (0—absent, 1—slight, 2—moderate, 3—severe). The inflammation process and damaged regions were graded as described previously [40]. The total histopathology score of each organ was calculated as an average of individual criteria scores. The uninfected tissue was used as a baseline score.

### 2.6. Transmission Electron Microscopy (TEM)

For transmission electron microscopy, animal tissues were fixed with 2.5% glutaraldehyde and 1% osmium tetroxide (OsO_4_) at 20 and 48 h after infection with *F. tularensis* subsp. *holarctica* LVS. Following the fixation, the samples were dehydrated by ethanol series, embedded in the epoxy resin (SPI Supplies, USA) and polymerized for 24–48 h at 60 °C, as previously described [41]. To define areas containing bacteria for ultrastructural examination, thin sections (0.5 µm) were stained with toluidine blue and scanned by light microscopy (Olympus IX51, Hamburg, Germany). Ultrathin sections (0.1 µm) were then cut, stained with lead citrate and uranyl acetate and examined by TEM (Zeiss 902A, Oberkochen, Germany). Each sample was investigated by transmission electron microscopy using the following criteria: cytosolic localization of bacteria, phagosomal localization of bacteria, and autophagosomal localization of bacteria.

### 2.7. Statistics

Statistical significances were determined using two-tailed Student’s *t* test. Statistical analyses were performed using Statistica (Statsoft) software version 12 or with GraphPad Prism version 6.0 software. In all cases, *p* < 0.05 were accepted as significantly different and were denoted by *. 

### 2.8. Ethics Statement

All the experimental procedures were in compliance with National guidelines and were approved by the Institutional Animal Care and Use Committee at Faculty of Medicine, University of Rijeka. All the animal experiments were approved by the Ministry of Agriculture, approval number HR-POK-016.

## 3. Results

### 3.1. Atg5 Exacerbates Infection in Vivo with F. tularensis LVS

The role of Atg5-dependent autophagy for survival of mice was investigated using Atg5-deficient mice and control mice. Mice were infected with 5 × 10^4^, 5 × 10^5^, 5 × 10^6^, 5 × 10^7^, or 5 × 10^8^ of the LVS intradermally. 

**Figure 1:**
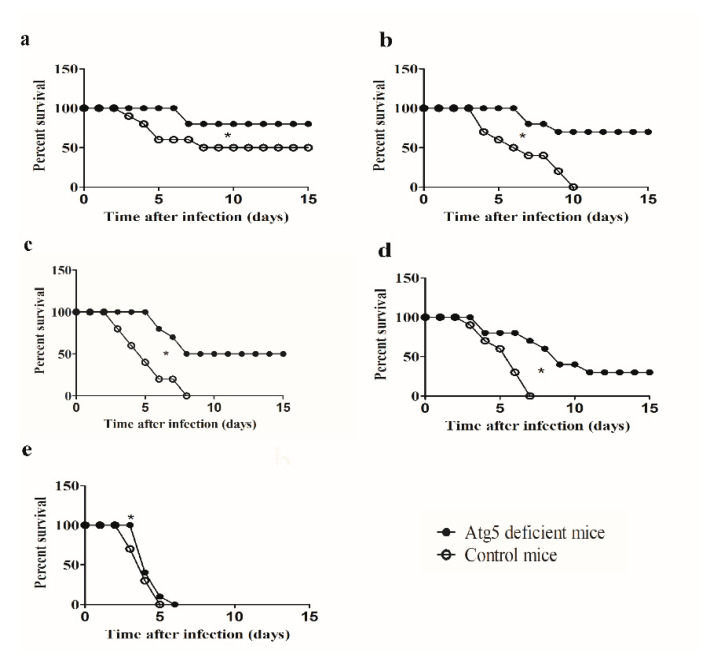
Survival of Atg5-deficient mice and control mice infected with the live vaccine strain (LVS) of *F. tularensis*. Groups of 10 mice were infected with (**a**) 5 × 10^4^ (**b**) 5 × 10^5^ (**c**) 5 × 10^6^ (**d**) 5 × 10^7^, or (**e**) 5 × 10^8^ of bacteria per mouse intradermally. Infected animals were monitored for 15 days. The Prism GraphPad5 software was used to generate graphs and calculate statistical values. * *p* < 0.05 was considered significant in comparison to the control mice.

After infection of Atg5-deficient mice with 5 × 10^4^ CFU/mouse, mice started to die at day 7 after infection (Figure 1A). However, the survival of Atg5-deficient mice during 15-day observation was 80%. In contrast, in the control group of mice infected with 5 × 10^4^ of the LVS 50% of mice survived, and the mean time to death (MTTD) was 5 days (*p =* 0.0006) (Figure 1a, Table 1). Thus, the infection dose of 5 × 10^4^ represented the 50% of lethal dose (LD_50_) for the control group of mice. The survival of Atg5-deficient mice infected with a dose of 5 × 10^5^ CFU/mouse was 70% (MTTD = 7.5 days), while in the control group of mice was 0% leading to death between 4 and 10 days post infection (MTTD = 6 days) (*p* < 0.0001) (Figure 1b, Table 1).

The intradermal infection of Atg5-deficient mice using the dose of 5 × 10^6^ CFU/mouse resulted in 50% survival during the 15 days observation (MTTD = 7 days) (Figure 1c, Table 1) Thus, for the Atg5-deficient mice, the LD_50_ was 5 × 10^6^ CFU/mouse (Figure 1c). In contrast, the survival rate of control mice infected with the LVS at the dose of 5 × 10^6^ CFU/mouse was 0%, and the mean time to death was 4.5 days (*p* < 0.0001) (Figure 1c, Table 1). The survival of Atg5-deficient mice after the dose of 5 × 10^7^ CFU/mouse was 30% during the 15-day observation period with the mean time to death of 6.5 days, while in the control group of mice survival was 0% and MTTD was 4 days (*p* < 0.0001) (Figure 1d, Table 1).

With the highest tested dose of bacteria (5 × 10^8^ CFU per mouse) in the group of control mice as well as Atg5-deficient, the survival rate was 0% (Figure 1e). The only difference in survival rate was observed at the third day of infection, whereas 100% of Atg5 mice were alive in contrast to 70% of survival in control group of mice (*p* = 0.0006) (Figure 1e). The mean time to death of Atg5-deficient mice after infection with 5 × 10^8^ CFU per mouse was 4 days and of control mice 3.5 days (Table 1).

Collectively, our results show that the intradermal infection of Atg5-deficient mice with the LVS at the doses of 5 × 10^5^ CFU/mouse, 5 × 10^6^ CFU/mouse and 5 × 10^7^ CFU/mouse resulted in 70%, 50% and 30% survival, respectively. In contrast, none of control mice survived these doses. Thus, Atg5-dependent autophagy increases the mortality of mice infected with *F. tularensis*.

### 3.2. Replication of F. tularensis in Vivo is Dependent on Atg5

To determine the role of the Atg5-dependent autophagy on the replication of the LVS in an in vivo model, the intracellular replication in the spleen, liver, and lung of the Atg5-deficient mice and the control mice were examined (*n* = 3). The mice were intradermally infected with the LVS with a dose of 5 × 10^4^ bacteria per mouse. At 6, 20, 48 and 72 h after infection, the spleen, liver, and lung were harvested from the infected mice and plated to determine the number of bacteria in the organs.

**Figure 2:**
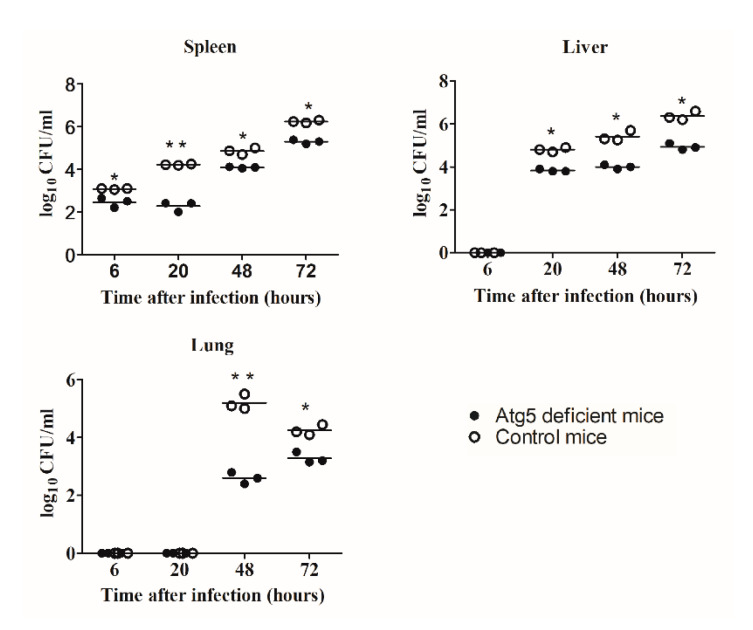
Growth kinetics of the LVS in the spleen, liver, and lungs of the Atg5-deficient and control mice. Each group of mice was infected intradermally with 5 × 10^4^ bacteria per mouse. To determine the bacterial loads in the lung, liver, and spleen, organs were harvest and homogenized. Three mice per group were used in this experiment. Mean values are indicated. * *p* < 0.05, ** *p* < 0.001 were considered significant in comparison to the control mice.

The LVS replicated efficiently in the spleen of the infected animals (Figure 2). At 6 h post infection (p.i.), the bacterial count in the spleen of the control group (1 × 10^3^ CFU/mL) was slightly higher than in the spleen of the Atg5-deficient mice (3 × 10^2^ CFU/mL) (*p* = 0.047) (*n* = 3) (Figure 2). There is a small difference between these two groups at 6 h p.i. in growth kinetics but we do not believe this has any biological relevance. However, by 20 h p.i. there was a statistically significant increase in the bacterial proliferation in the spleen of the control mice (3 × 10^4^ CFU/mL) compared to the Atg5-deficient mice (2 × 10^2^ CFU/mL) (*p* < 0.001) (*n* = 3) (Figure 2). Furthermore, the number of bacteria in the spleen of the control mice reached 2 × 10^6^ CFU/mL by 72 h after infection, while the number of bacteria in the spleen of the Atg5-deficient mice was 2 × 10^5^ CFU/mL (*p* = 0.013) (*n* = 3) (Figure 2).

The LVS was not detected in the liver of the infected mice at 6 h after infection (Figure 2). At 20 h p.i., the bacterial numbers in the liver of the Atg5-deficient mice was statistically reduced compared to the control group (*p* = 0.001) (*n* = 3) (Figure 2). The same phenomenon was observed at 48 h after infection. The highest number of bacteria was observed at 72 h after infection in the liver of the control mice (2 × 10^6^ CFU/mL), while the number of the bacteria in the liver of the Atg5-deficient mice was 2 × 10^4^ CFU/mL, the same as the initial dose of infection (*p* = 0.003) (*n* = 3) (Figure 2).

The LVS was not detected in the lung of the infected mice at 6 and 20 h after infection (*n* = 3) (Figure 2). By 48 h p.i., the bacterial count in the lung of the control mice was 2 × 10^5^ CFU/mL (*n* = 3) (Figure 2). In comparison to the control group, the number of the LVS in the lung of Atg5-deficient mice was significantly reduced at 48 h p.i. to 4 × 10^2^ CFU/mL (*p* < 0.0001) (*n* = 3) (Figure 2). At 72 h after infection, the number of the LVS in the lung of the control mice declined to 2 × 10^4^ CFU/mL, as well as the bacterial count in the lung of Atg5-deficient mice declined to 2 × 10^3^ CFU/mL (*p* = 0.005) (*n* = 3) (Figure 2). 

Our results show that the bacterial replication was significantly reduced in all investigated organs of the Atg5-deficient mice. 

### 3.3. The LVS Causes Less Severe Pathological Changes in Organs of Atg5-Deficient Mice

Since the LVS showed a reduced intracellular replication in the organs of the Atg5-deficient mice, the histolopathogical changes in the spleen, liver, and lung of the infected mice were examined in comparison to the control mice. Mice were infected intradermally with the LVS in a dose of 10^4^ bacteria per mouse. The histopathological changes of each organ were observed at 20, 48, and 72 h after infection and the analysis was conducted by use of a light microscope (Olympus IX51, Hamburg, Germany). The histopathology score was an average of the scores of the individual criteria for each organ. 

**Figure 3:**
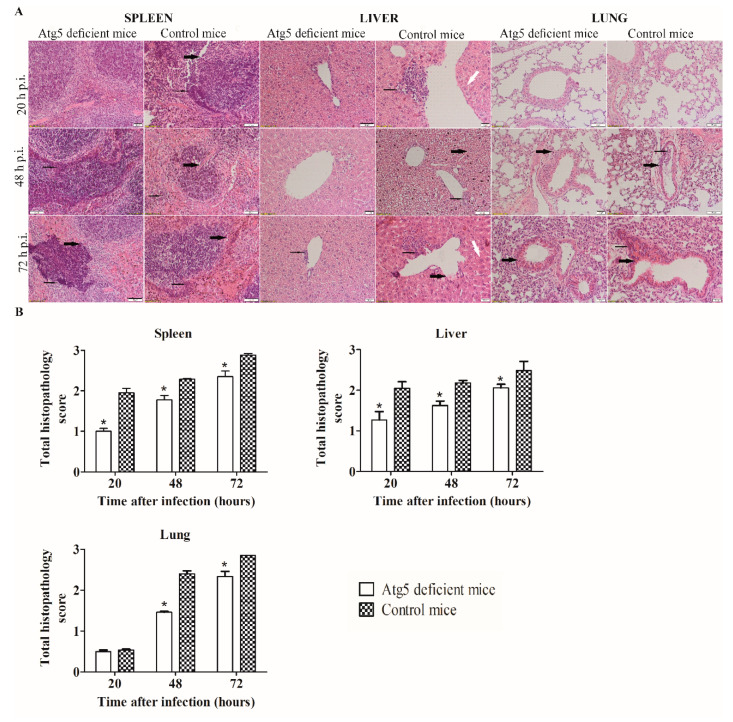
Histopathology and histopathology scoring of spleen, liver and lung tissue. At 20, 48, and 72 h after intradermal infection of the Atg5-deficient and control mice with the LVS, spleen, liver, and lung tissue sections were stained with hematoxylin and eosin (H&E). The experiments were conducted in triplicate using three mice per group. (**A**) A thin black arrow shows inflammatory infiltration, a thin white arrow shows the vacuolation of hepatocytes in the liver tissue, and a thick black arrow shows the destruction of the tissue parenchyma. Scale bars either 10 µm or 50 µm. (**B**) Twenty random high-powered fields were assessed. The spleen was scored for destruction of architecture of white pulp, red pulp and capsule (0—absent, 1—slight, 2—moderate, 3—severe). Liver was scored for inflammatory infiltrates and hepatocyte vacuolation (0—absent, 1—slight, 2—moderate, 3—severe). Lung tissue was scored for the alveolar and bronchial destruction, and infiltration of mononuclear cells (0—absent, 1—slight, 2—moderate, 3—severe). The error bars represent SD. * *p* < 0.05 was considered significant in comparison to control mice.

At 20 h after infection, the destruction of the architecture of the red pulp, white pulp, and the capsule of the spleen tissue of the control mice was moderate (Figure 3A,B). The histopathological changes in the spleen of the Atg5-deficient mice infected with the LVS were less severe in comparison to the control group (*p* = 0.008) (Figure 3A,B). Furthermore, the most severe splenic histopathological changes were observed in the control mice at 72 h after infection, whereas there were no histopathological changes in the spleens of the Atg5-deficient mice (Figure 3A,B).

Within the liver parenchyma of the control mice, at all time points, there were numerous areas of inflammatory infiltration and hepatocyte degeneration. In contrast, less severe histopathologic changes were observed within the liver of the Atg5-deficient mice (*p* < 0.05) (Figure 3A,B).

At 20 h after infection, there were only minor differences of the cellular composition observed in the lungs between the two groups of mice (Figure 3A,B). At 48 h after infection, infiltration of mononuclear cells within the peribronchiolar spaces, bronchiole and alveoli were present in the control group. In addition, there was severe lung parenchyma destruction (Figure 3A,B). In contrast, the histopathological changes in the Atg5-deficient mice were less severe (*p* = 0.003) (Figure 3A,B). At 72 h after infection, the histopathological changes of the control group were similar to those at 48 h. In the lung tissues of the Atg5-deficient mice, the infiltration of mononuclear cells and the parenchyma destruction were present to a greater extent at 72 h than at 48 h after infection; however, with less intensity in comparison to the control mice (*p* = 0.027) (Figure 3A,B).

Altogether, we conclude that the histopathological changes in the spleen, liver, and lung tissues were less severe in Atg5-deficient mice in comparison to the control mice. 

### 3.4. Vacuolar Localization of Bacteria in Tissues of Infected Atg5-Deficient Mice

**Figure 4:**
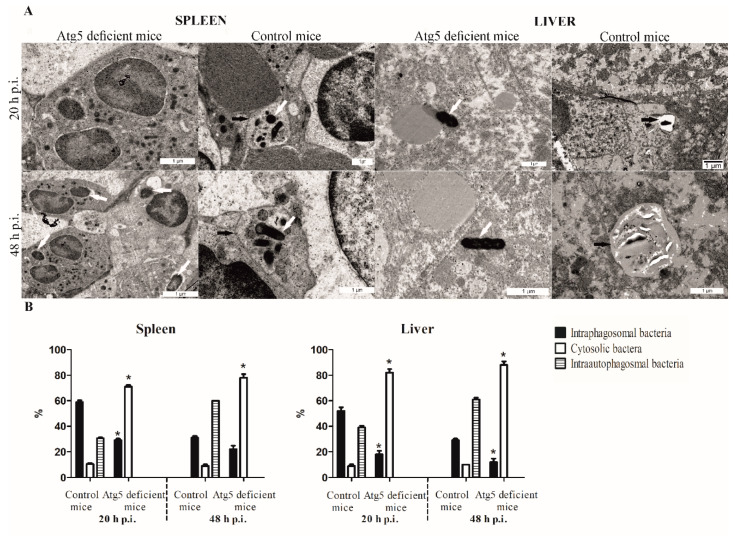
Transmission electron micrographs of spleen and liver tissues of the Atg5-deficient mice and control mice. (**A**) At 20 and 48 h after infection of mice with the LVS, tissues were excised, fixed with glutaraldehyde and processed for electron microscopy. Black arrows show autophagosomal vacuoles and white arrows show bacteria. (**B**) Quantitative analyses of the intact bacteria-containing vacuole. The integrity of the intact vacuoles was determined by electron microscopy counting at least 100 bacteria for each sample using following criteria: cytosolic localization of bacteria, phagosomal localization of bacteria, and autophagosomal localization of bacteria. The error bars represent SD. * *p* < 0.05 was considered significant in comparison to control mice.

The location of the bacteria within the cells in the tissues of the spleen, liver, and lungs in the control mice and the Atg5-deficient mice was determined by transmission electron microscopy at 20 and 48 h after the infection. At 20 h post infection, 11% of the LVS bacteria were found in the cytosol of the control mice splenocytes which was significantly different from the Atg5-deficient mice where 71% of bacteria were in the cytosol of the splenocytes (*p* = 0.0001) (Figure 4). Further, 30.5% of bacteria were within an autophagic vacuole (double-membrane vacuole) in the splenocytes in the control mice in comparison to the bacteria in the splenocyte within the splenic tissues of the Atg5-deficient mice, where 0% of the bacteria were in the autophagic vacuole (*p* = 0.0001) (Figure 4). At 48 h after infection, 60% of the bacteria were in the autophagic vacuole within the splenic tissue. In contrast, 0% of bacteria were in the autophagic vacuole in the spleen of the Atg5-deficient mice (*p* < 0.0001) (Figure 4). The bacteria were present mainly in the macrophages in the spleen tissue (Figure 4).

In the liver tissue, 20 h after infection of Atg5-deficient mice and control group of mice, 82% and 9% of the bacteria were in the cytosol of the hepatocytes, respectively (*p* = 0.0001) (Figure 4). At 48 h, 61% of the bacteria were localized within the autophagic vacuoles in the control mice, whereas the corresponding percentage in Atg5-deficient mice was 0% (*p* = 0.0001) (Figure 4). Most of the cells from the ultrastructural analyses were hepatocytes (Figure 4).

We conclude that Atg5 is responsible for the processing of the bacteria into the autophagic vacuole which supports the replication of the LVS in the liver and spleen tissue.

## 4. Discussion

*F. tularensis* is a highly infectious, facultative intracellular pathogen that causes the zoonotic disease tularemia [25]. *Francisella* escapes the phagosome rapidly after the infection and reaches the host cell cytosol where the bacteria undergo an extensive replication [28,42]. Once cytosolic, the bacteria become a target of the autophagy-mediated process [3]. Autophagy, a key innate immune response, is an efficient mechanism to degrade intracellular pathogens [8]. Previous studies have shown that some pathogens, such as *Mycobacterium tuberculosis*, *Streptococcus pyogenes,* and *Salmonella enterica*, are eliminated by the autophagic pathway [4,13,43]. However, several pathogens take advantage of the autophagy mechanism to survive and replicate. For example, *Coxiella burnetii* exploits the autophagic process to survive and proliferate within the cells. It was shown that *C. burnetii* replication increases when autophagy is induced [44,45]. Essential for the formation of canonical autophagosomes are proteins termed Atg proteins, such as Atg5, and Atg6 (Beclin 1) [46,47]. 

The mechanism of the autophagy in the pathogenesis of tularemia has been studied extensively in vitro. A previous study on mouse embryonic fibroblasts has shown that autophagy provides nutrients to support the replication of *F. tularensis* during infection by an Atg5-independent autophagy pathway [38]. Interestingly, after the inhibition of autophagy, the bacteria can still multiply due to an excess of amino acids or pyruvate, independent of Atg5 [38]. In this context, another study demonstrated that interference of *Francisella* with autophagic recognition ensures its proliferation and survival [11]. However, a recent study on the Atg5-deficient BMMs and a *F. tularensis* strain lacking the surface polysaccharide O-antigen showed that the O-antigen is a shield against autophagic recognition via Atg5-dependent autophagy [39]. Further, it has been shown that the autophagy mechanism in mice is identical to the one in human macrophages [39]. Future studies need to clarify these contradictory results. In contrast to a preponderance of in vitro studies on *F. tularensis*, the role of the autophagy pathway in vivo has not been documented.

Several recent studies have demonstrated the importance of in vivo experiments to fully understand the complex roles of the autophagy pathways in vivo. Previous studies have shown that Atg5 is required for in vivo resistance to *Listeria monocytogenes* and *Toxoplasma gondii*, and is essential for the expression of cellular immunity to these intracellular bacteria [48]. On the other hand, pathogens such as *Mycobacterium tuberculosis* are one of the first pathogens recognized as a target for elimination by the autophagy pathway. A study using Atg5-deficient mice showed that autophagy suppresses the replication of *M. tuberculosis* and prevents an excessive inflammatory reaction in the host [4]. Additionally, it was demonstrated that the loss of Atg5 in monocyte-derived cells and neutrophils led to the sensitization of mice to *M. tuberculosis* associated with loss of their weight, uncontrolled tissue damage and progression of disease [49]. These findings indicate that Atg5 has a unique role in the protection against *Mycobacterium* infection in vivo [4,49]. 

Here, we explored the role of Atg5 for the *F. tularensis* infection in vivo, using mice specifically deficient for Atg5 in myeloid cells. Our study showed that intradermal infection of Atg5-deficient mice with the LVS resulted in a higher survival rate than in the wild-type mice. Furthermore, bacterial burdens in organs of Atg5-deficient mice were significantly lower and the infection caused less severe histopathological changes within the organs in comparison to wild-type mice. Additionally, the ultrastructural analyses of the organs of infected wild-type mice and Atg5-deficient mice showed that Atg5 is responsible for the localization of *Francisella* within the autophagic vacuole that supports bacterial proliferation. Although the intranasal infection was not addressed in this study, we assume that intranasal infection will result in a similar outcome. It would be interesting to investigate the role of lung epithelial cells after intranasal infection in future studies.

Collectively, we showed for the first time that the Atg5-dependent autophagy supports the efficient intracellular growth of *F. tularensis* LVS and affects the pathology of the tissues in vivo. The experiment regarding the mechanism behind the immune response is a subject of our current studies. Since we only have preliminary results, it is too early to speculate on the role of Atg5 in acquired immunity. Future research on Atg5 functions during the pathogenesis of tularemia should be focused on the elucidation of the inflammatory response regulated by autophagy to gain the much-needed information about this important pathway.

## Figures and Tables

**Table 1:** Mean time to death (MTTD) of Atg5-deficient mice and control mice infected with *F. tularensis* LVS with 5 × 10^4^, 5 × 10^5^, 5 × 10^6^, 5 × 10^7^, or 5 × 10^8^ of bacteria per mouse intradermally.

Infection Dose (CFU/mL)	MTD of Atg5-Deficient Mice (Days)	MTD of Control Mice (Days)
5 × 10^4^	7	5
5 × 10^5^	7.5	6
5 × 10^6^	7	4.5
5 × 10^7^	6.5	4
5 × 10^8^	4	3.5
